# Aluminum in Bottom Sediments of the Lower Silesian Rivers Supplying Dam Reservoirs vs. Selected Chemical Parameters

**DOI:** 10.3390/ijerph182413170

**Published:** 2021-12-14

**Authors:** Magdalena Senze, Monika Kowalska-Góralska, Katarzyna Czyż, Anna Wondołowska-Grabowska, Joanna Łuczyńska

**Affiliations:** 1 Department of Limnology and Fishery, Institute of Animal Breeding, Wrocław University of Environmental and Life Sciences, Poland, ul. Chełmońskiego 38c, 51-630 Wrocław, Poland; monika.kowalska-goralska@upwr.edu.pl; 2 Department of Sheep and Fur Animals Breeding, Institute of Animal Breeding, Wrocław University of Environmental and Life Sciences, ul. Kożuchowska 5A, 51-631 Wrocław, Poland; katarzyna.czyz@upwr.edu.pl; 3 Institute of Agroecology and Plant Production, Wrocław University of Environmental and Life Sciences, Grunwaldzki Sq. 24A, 50-363 Wrocław, Poland; anna.wondolowska-grabowska@upwr.edu.pl; 4 Faculty of Food Sciences, University of Warmia and Mazury in Olsztyn, ul. Plac Cieszyński 1, 10-726 Olsztyn, Poland; jlucz@uwm.edu.pl

**Keywords:** bottom sediments, aluminum, rivers, water, indicators: MPI, I_geo_, C_f_, DC, CF

## Abstract

The study was carried out on sediments collected from three rivers: Nysa Szalona, Strzegomka and Bystrzyca flowing in southwestern Poland. The content of Al in sediments and in bottom water was determined in relation to chemical conditions. The study was carried out in a four-year cycle, during spring and autumn. The aim of the study was to determine the level and accumulation of aluminum in sediments of rivers supplying dam reservoirs storing water for consumption. The sediments studied were mineral in nature, with neutral pH and moderate sulfate content. The level of Al and heavy metals in the sediments was the highest in the Nysa Szalona River and the lowest in the Strzegomka River, which was also evident in the concentration factor (CF). In terms of season, higher Al contents were recorded in sediments in autumn than in spring, which was also reflected in the concentration factor (CF). Along the course of the river, a gradual decrease in Al levels was observed in successive tributaries in the Nysa Szalona and Strzegomka Rivers, while there was no apparent regularity for the Bystrzyca. Against this background, a comparison of extreme sites below the springs and at the reservoir outlet shows that values were higher in the Nysa Szalona below the springs, and lower in the Strzegomka and Bystrzyca below the reservoir outlet. The general picture of Al and heavy metal loading of the studied sediments shows the lowest loading for the Strzegomka, only the enrichment factor (EF) was the lowest for the Nysa Szalona: metal pollution index (MPI)—S < B < NS, contamination factor (C_f_)—S < B < NS, degree of contamination (DC)—S < NS < B, EF—NS < B < S, geoaccumulation index (I_geo_)—S < B < NS, CF—S < NS < B. There was no effect of catchment size and river length on Al levels in sediments.

## 1. Introduction

Surface water bodies act as filters by retaining pollutants flowing into them from catchment areas. Accumulation of pollutants takes place in the tissues of living organisms and in bottom sediments, which under stable conditions are accumulated and do not pose a threat to the aquatic environment. However, they can be released into the water during floods, volcanic eruptions, earthquakes, or human construction work in the catchment area or in the riverbed itself. In flowing waters of mountainous sections, sediment deposition takes place to a rather small extent. Too fast water current does not allow to capture organic and mineral matter. The accumulation of its scanty amount in the mountainous section of the river in summer and autumn together with spring thaws is washed out when large masses of water flow down the river often with great force. The formation and maintenance of sediment is possible only in the lowland section, when the water current becomes slower and the sedimentation process is visible. The bottom sediment that accumulates in the river bed acts as a kind of pollution store. It contains dead plant and animal remains, but also chemical compounds flowing with the water. River beds are subject to modifications, most often carried out in sub-mountain areas and in towns. Regulation covers not only the river bed, but also the banks. Rivers are excessively straightened, water flows fast, which does not favor the accumulation of bottom sediments. Similar hydrotechnical modifications were applied to the studied rivers of Lower Silesia: Nysa Szalona, Strzegomka and Bystrzyca and their tributaries, which are the main source of supply for dam reservoirs. These rivers are particularly important because water from them is obtained for the Lower Silesian urban agglomerations. These rivers differ, among others, in their length and catchment structure [1,2,3,4].

The presence of organic and mineral compounds in the bottom sediments in different proportions is usually recorded. The level of mineral compounds in water reservoirs depends on their content in the catchment but can also be the result of their release from bottom sediments. Aluminum compounds occupy a considerable amount in the sediments, which under natural conditions do not have a negative impact on the life in the reservoir and on the quality of the water obtained from it [5,6,7]. Aluminum, along with oxygen and silicon, is one of the main building blocks of the earth’s crust, accounting for 7.91% of the mass of the lithosphere, and its presence is recorded in all rocks. This element is contained in 250 minerals, 40% of which are aluminosilicates: orthoclase, albite, anorthite, muscovite, kaolinite. Depending on the type of rock, the content of aluminum is variable. The greatest amount occurs in magmatic and sedimentary rocks, in which the average level is about 7.50–8.00%, including: ultramafic magmatic rocks (0.45%), alkaline (8.76%), intermediate (8.85%), acidic (7.70%), and in sedimentary rocks: clay (8.00%), sands (2.50%) and limestone (0.42%). Aluminum occurs mostly in the form of Al^+3^ cation and shows affinity to oxygen bonds [8,9,10,11,12].

Aluminum is an amphoteric element, meaning it has both an acidic and a basic character. The pH is of decisive importance for the release of aluminum from soil and water. Therefore, in surface waters with extreme values of pH, it can occur in elevated concentrations. This condition may be due to natural causes or as a result of anthropogenic activities [8,11,12,13]. The presence of aluminum in water is not indispensable, and it used to be considered a completely harmless element for aquatic organisms. However, at the turn of the 20th century, the first reports of its toxicity appeared [14]. In the 1950s, it was proved that aluminum from acidified soil solutions penetrates surface water. It may therefore be present in drinking water especially when it is collected in areas of acidic rocks and soils [10,11,15,16].

In addition, water can be enriched with aluminum as a result of water pipes contamination. Aluminum is also used as a water conditioner for drinking water and in wastewater treatment processes [11]. Aluminum levels in the ambient air reflect the natural dustiness that increases in urbanized areas (coal burning, metallurgical industries) [10,16].

Because aluminum occurs in bottom sediments as complexes of organic compounds, fluorides, and sulfates, it is present in water at low concentrations—less than 1.0 mg·dm^−3^. Aluminum is readily absorbed by bottom sediments in the form of metastable compounds. As water acidity increases, it can become activated. Only a change in pH caused by, among others, acid precipitation can dissolve minerals and release aluminum into the water. When the pH is acidic, elevated concentrations of aluminum are recorded, reaching up to 5 mg·dm^−3^ [6,8,10,16,17].

The natural level of aluminum in water has increased significantly in many cases with the development of civilization. Aluminum is also to a higher degree available to living organisms and perceived as a natural components is treated as pollution [7,9,18,19,20].

The aim of the study was to determine the level and accumulation of aluminum in the bottom sediments of rivers supplying dam reservoirs that store water for drinking purposes against the background of chemical conditions.

## 2. Material and Methods

### 2.1. Research Area

**Figure 1:**
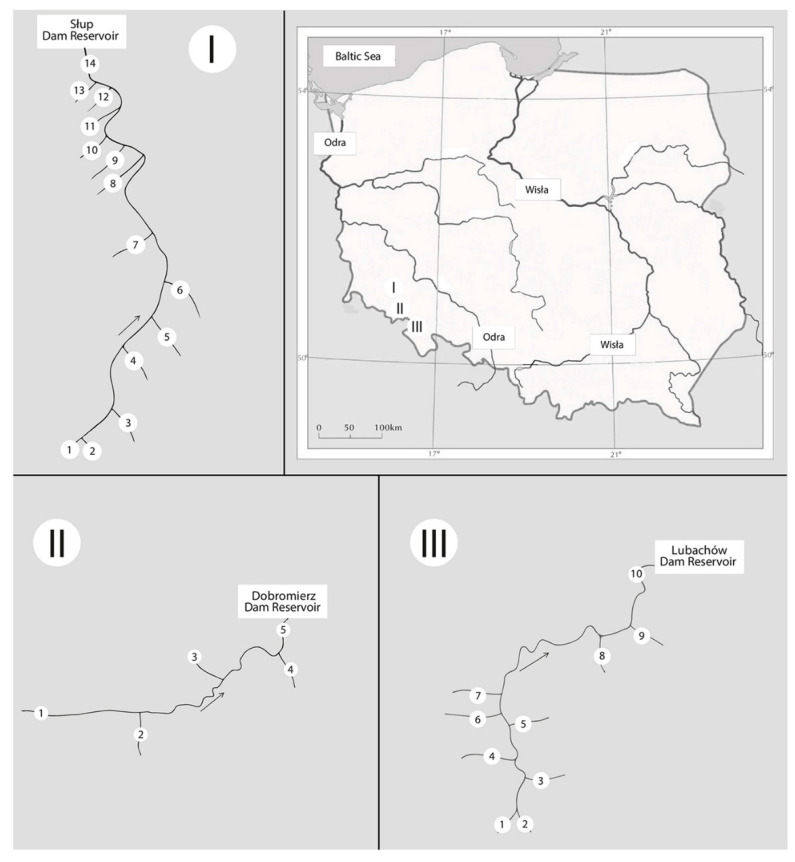
Location of the study area, **I**—Słup Reservoir—research sites on the Nysa Szalona River and its tributaries, **II**—Dobromierz Reservoir—research sites on the Strzegomka River and its tributaries, **III**—Lubachów Reservoir—research sites on the Bystrzyca River and its tributaries.

The study covered the following areas in south-west Poland: N 50°38′10.1652″–N 51°4′31.7745″ and E 16°3′54.4715″–E 16°25′1.4097″ (Figure 1). The study included three Lower Silesian rivers belonging to the Oder basin—the Nysa Szalona, the Bystrzyca and the Strzegomka and their tributaries.

Nysa Szalona—third-order river, right-bank tributary of the Kaczawa River, length 51.00 m, catchment area 443.10 km^2^, springs at 628 m above sea level (Pustelnik Mountain). The dam reservoir Słup was built in 1984 on the river in 8.20 km—its function is retention (flood wave reduction) and municipal water supply for the Legnica region, it is a lowland reservoir (165–257 m asl). The size of the catchment above the reservoir—374.81 km^2^. The catchment area of the river—agricultural and forest areas, grasslands, sewage treatment plants (Wolbromek, Jawor), aggregate mine, expressways, bigger towns Bolków, Jawor. Right-bank tributaries above the reservoir—Ochodnik, Sadówka, Czyściel, Parowa, Kocik; left-bank tributaries—Ochodnik, Sadówka, Czyściel, Parowa, Kocik. Soils in the catchment—podzolic, brown podzolic, alluvial soils [2,3,4,21].

Strzegomka—second-order river, left-bank tributary of the Bystrzyca River, length 74.70 km, catchment area of 555.00 km^2^, springs at 692.00 m above sea level (Trójgarb Peak). The dam reservoir Dobromierz was built in 1988 on the river in 62.00 km—its function is retention (reduction of flood waves) and municipal water supply for region of Swiebodzice, it is a lowland-highland reservoir (300–423 m asl). The catchment area above the reservoir is 70.32 km^2^. The catchment area is agricultural land, grasslands, loose rural buildings, unorganized sewage collection, Stare Bogaczowice is a major town. Right-bank tributaries above the reservoir—Polska Woda, Czyżynka; left-bank tributary—Sikorka. Soils in the catchment—podzolic, brown, alkaline, acidic soils [1,4,21].

Bystrzyca—second-order river, left-bank tributary of the Oder River, length 95.20 km, catchment area 1767.80 km^2^, springs at an altitude of 618.00 m above sea level (Suche and Sowie Mountains). The dam reservoir Lubachów was built in 1918 on the river in 78.00 km—its function is retention (flood wave reduction), energy, water supply and municipal for Dzierżoniów region, it is an upland reservoir (400–500 m asl). The size of the catchment area above the reservoir—130.69 km^2^. The river catchment—agricultural and forest areas, grasslands, a sewage treatment plant and a waste dump (Jugowice), larger towns: Głuszyca, Jugowice, Zagórze Śląskie. Right-bank tributaries above the reservoir—Złoty Potok, Kłobia, Potok Marcowy Duży, Jaworzynik, Walimianka; left-bank tributaries—Otłuczyna, Złota Woda, Rybna. Soils in the drainage basin—podzolic, brown, leached soils, deluvial deposits [4,21].

### 2.2. Material

The research material consisted of bottom sediments and water collected from the same sites from the rivers: Nysa Szalona, Strzegomka and Bystrzyca and their tributaries (50 m before their confluence with the main rivers) (Figure 1). Samples from the main rivers were collected below the sources of each river and at their mouths in dam reservoirs (Nysa Szalona–Słup Reservoir, Strzegomka–Dobromierz Reservoir, Bystrzyca–Lubachów Reservoir). The study was conducted over a four-year period (2015–2018). Material was collected in spring (April/May) to capture the impact of winter melt, and in autumn (September/October) mainly the impact of agricultural management on water quality.

I —Research sites—Nysa Szalona River [4]:
  1. Nysa Szalona below the springs in Domanów (N 50°51′38.8261″; E 16°3′54.4715″)—upland silicate stream with coarse-grained substrate—western, type 4, SWB * status—natural
  2. Kocik (N 50°52′15.4891″; E 16°4′5.9042″)
  3. Ochodnik (N 50°53′37.1718″; E 16°5′59.7672″)
  4. Sadówka (N 50°55′58.609″; E 16°10′11.3627″)
  5. Czyściel (N 50°57′49.4252″; E 16°13′57.6982″)
  6. Radynia (N 50°58′56.648″; E 16°14′13.9202″)
  7. Nysa Mała (N 51°0′10.455″; E 16°12′26.0825″)—Upland carbonate stream with coarse-grained substrate, type 7, SWB* status—natural
  8. Puszówka (N 51°2′30.3945″; E 16°11′39.425″)
  9. Jawornik (N 51°2′57.6884″; E 16°10′52.4584″)
  10. Księginka (N 51°3′17.4033″; E 16°10′11.2082″)
  11. Starucha (N 51°4′31.7745″; E 16°9′17.7528″)—Upland silicate stream with fine-grained substrate—western, type 5, SWB* status—natural
  12. Rowiec (N 51°4′22.844″; E 16°8′27.5419″)
  13. Męcinka (N 51°4′29.2507″; E 16°7′28.5247″)
  14. Nysa Szalona mouth to the Słup Reservoir (N 51°4′29.2507″; E 16°7′28.5247″)—Small upland silicate river—western, type 8, SWB* status—artificial.

* SWB—surface water body

II —Research sites—Strzegomka River [4]:
  1. Strzegomka below the springs in Nowe Bogaczowice (N 50°50′14.5978″; E 16°7′49.845″)—Upland silicate stream with coarse-grained substrate—western, type 4, SWB * status—artificial
  2. Polska Woda (N 50°52′48.0601″; E 16°11′56.4194″)
  3. Sikorka (N 50°51′47.2613″; E 16°13′21.3918″)
  4. Czyżynka (N 50°52′15.8303″; E 16°14′29.8332″)
  5. Strzegomka mouth to the Dobromierz Reservoir (N 50°53′11.1994″; E 16°13′58.4707″)—Upland silicate stream with coarse-grained substrate—western, type 4, SWB* status—artificial

* SWB—surface water body

III —Research sites—Bystrzyca River [4]:
  1. Bystrzyca river below the springs in Wrześnik (N 50°38′10.1652″; E 16°24′5.7915″)—Upland silicate stream with coarse-grained substrate—western, type 4, SWB * status—artificial
  2. Złoty Potok (N 50°38′29.3697″; E 16°24′41.0163″)
  3. Kłobia (N 50°40′9.374″; E 16°23′27.0131″)
  4. Otłuczyna (N 50°40′36.2015″; E 16°22′46.8444″)
  5. Potok Marcowy Duży (N 50°41′5.2762″; E 16°22′32.3218″)
  6. Złota Woda (N 50°41′4.2973″; E 16°22′11.0015″)
  7. Rybna (N 50°41′49.8085″; E 16°21′58.1784″)
  8. Jaworzynik (N 50°43′25.8799″; E 16°23′56.5218″)
  9. Walimianka (N 50°43′49.9381″; E 16°24′15.0612″)
  10. Bystrzyca mouth to the Lubachów Reservoir (N 50°45′5.8065″; E 16°25′1.4097″)—Upland silicate stream with coarse-grained substrate—western, type 4, SWB* status—artificial

* SWB—surface water body

### 2.3. Analytical Methods

The surface layer of bottom sediments (to a depth of 10 cm) was collected with an Ekman sampler (size 15 cm × 15 cm) (HydroBios, Germany) directly into cloth bags, dried at room temperature to air-dryness, crushed in a mortar and sieved through a sieve with a mesh diameter of 2 mm [22].

A 2 g air-dry and homogenized sample was weighed in an HP-500 Teflon dish (CEM Corporation, Matthews, NC, USA). After adding 10 cm^3^ HNO_3_:HClO_4_ (3:1) (Sigma-Aldrich, Poznań, Poland), the samples were left at room temperature for 24 h. They were then placed in a Mars 5 microwave digestion oven (CEM Corporation, Matthews, NC, USA) and subjected to a 3-stage mineralization. After cooling to room temperature, the mineralizates were transferred to test tubes and diluted with distilled water to 25 cm^3^ [23]. Aluminum and heavy metal levels were determined using a Spectra AA-110/220 (Varian, Melbourne, Australia) [24,25,26].

Basic chemical properties were determined:

- pH of sediments (for sediments in potassium chloride) by potentiometric method using pH-meter PH-207 (Slandi, Michałowice, Poland) [27]
- mineral and organic compounds in sediments by weight [28]
- sulfates in sediments by nephelometry [29]
- total aluminum in sediments by electrothermal atomic absorption spectrometry (ETAAS) (Varian, Melbourne, Australia) [30]
- lead, copper, nickel, zinc, cadmium, iron, manganese in sediments by flame atomic absorption spectrometry (FAAS) (Varian, Melbourne, Australia) [31]

A total of 232 bottom sediment samples were collected. Results are given in mg·kg^−1^ for bottom sediments with respect to dry weight. The results of the study were verified using certified reference materials LKSD-2—Canadian Certified Reference Materials Project (CANMET).

### 2.4. Analysis of the Results

Analysis of results was performed using Microsoft Office Excel 2019 and Statistica 13.0. software (StatSoft Poland, Krakow, Poland).

The assessment of the state of sediments contamination with aluminum and heavy metals was carried out using several indices. An average value of 0.65 was adopted for Al, 20,000.00 for Fe and 500.00 for Mn for water sediments as reported for Wałbrzych and its surroundings [32]. For metals, the following values were taken as the sediment geochemical background according to Bojakowska and Sokołowska [33]: Cd: 0.50, Cu: 6.00, Ni: 5.00, Pb: 10.00, Zn: 48.00.

The results for water used in this paper have been published by Senze et al. [34].

- aluminum concentration factor (CF) in sediments (Equation (1)) [35]

CF = C_O_/C_W_ (1)

C_O_—aluminum content in bottom sediment, C_W_—aluminum concentration in water

- metal pollution index (MPI) for aluminum and heavy metals in relation to the degree of contamination (Equation (2)) [36] (Table 1).

MPI = (Cf_1_ x Cf_2_…Cf_n_)^1/n^ (2)

Cf_1_, Cf_2_… Cf_n_—metal concentration in subsequent samples.

- contamination factor by aluminum and heavy metals (C_f_) in relation to the level of contamination (Equation (3)) [37] (Table 2)

(3) $$C_{f}=C/\mathrm{Co}$$

C—mean concentration of aluminum in sediment, C_o_—geochemical background.

- degree of contamination (DC) with aluminum and heavy metals in relation to the level of contamination (Equation (4)) [37] (Table 2)

DC = Σ C_f_ (4)

C_f_—contamination factor

- metal enrichment factor (EF) (Equation (5)) [38] (Table 3)

EF = (Me/Al)_sample_ /(Me/Al)_background_ (5)

Me—particular metals, Al—aluminum.

- geoaccumulation index (*I_geo_*) and standard of geochemical purity classes of bottom sediments (Equation (6)) [33,38] (Table 4)

(*I_geo_*) = log_2_ [M_o_/(1.5·M_t_)] (6)

M_o_—concentration of metal in sediment, M_t_—geochemical background of metal.

### 2.5. Statistical Analysis of the Results

Analysis of the results was performed using Microsoft Office Excel 2019 and Statistica 13.0. Calculations were performed using R version 3.6.0. Shapiro-Wilk text was performed to verify normality of the distribution. Spearman correlations were used due to the distribution of samples. Spearman correlation was calculated in Statistica software. All statistically significant differences were calculated at *p* < 0.05. Due to the data being defined as having a non-normal distribution, the Kruskal–Wallis test with post-hoc analysis was used. An attempt was made to determine the value allowing the data to be divided into two groups differing in a statistically significant manner.

The PCA test using r-statistics was applied in order to visualize the differences between the groups (RStudio Version 1.1.442—©2009–2018, RStudio, Inc., Boston, MA, USA). It was performed on the basis of all data and presented: differences in the parameters of the examined rivers depending on season of research, years or section of rivers. Principal component analysis (PCA) is a technique for reducing the dimensionality of such datasets, increasing interpretability but at the same time minimizing information loss [39]. PCCA was performed using Statistica 13.0 software (StatSoft Poland, Krakow, Poland). PCCA (phylogenetic canonical correlation analysis) is a new program for canonical correlation analysis of multivariate continuous data from biological species [40].

## 3. Results and Discussion

Table 5 shows chemical properties of the bottom sediments and water. The sediments had a pH ranging from slightly acidic to slightly alkaline (pH 6.07–8.66), and the overall image for all rivers was close to neutral. The pH of the water was more alkaline than that of the sediments [34].

The sediments were dominated by mineral material (13.50–99.98%), with the highest average recorded in the Strzegomka River and the lowest in the Bystrzyca River. Occasionally, larger amounts of organic matter were found, which may have been the result of a single runoff from the catchment.

Sulfate levels in sediments ranged from 1.42 mgSO_4_·kg^−1^ to 73.16 mgSO_4_·kg^−1^. Mean sulfate content in the Nysa Szalona River was nearly twice as high as in the Bystrzyca and Strzegomka Rivers. Most probably higher sulfate level was related to higher (though sporadic) amounts of organic matter present in the river. In water, the level reached 55.68 mgSO_4_·dm^−3^, and the mean concentration was more even among the three rivers [34].

Heavy metal contents in water and sediments were the highest in the Nysa Szalona River and the lowest in the Strzegomka River [34]. In the Strzegomka River, for both water and sediments, the series of increasing values was as follows: Cd < Cu < Pb < Ni < Zn < Mn < Fe. For the Nysa Szalona and Bystrzyca Rivers, the inner part of the series was variable, with the lowest amount for cadmium and the highest for iron in both components.

**Figure 2:**
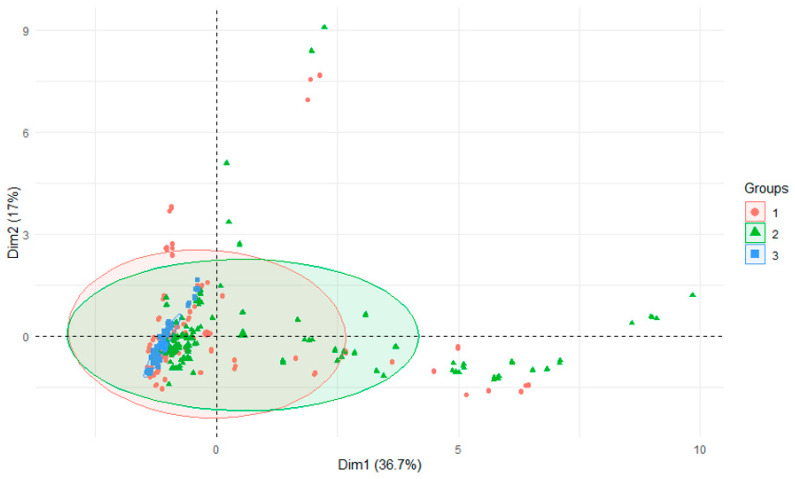
PCA plot 2D showing clustering of rivers across 29 sites and 3 features (1—Bystrzyca, 2—Nysa Szalona, 3—Strzegomka).

The range of aluminum content in bottom sediments varied from 13.46 mgAl·kg^−1^ to 96,260.32 mgAl·kg^−1^, with the range for water being 0.0034–0.6020 mgAl·dm^−3^ (Table 6) [34]. The lowest amounts of aluminum were found in the sediments of the Strzegomka River and its tributaries (mean 164.33 mgAl·kg^−1^). Higher amounts were found in the Nysa Szalona River (12,239.84 mgAl·kg^−1^), and the highest averaged 17,332.30 mgAl·kg^−1^ in the Bystrzyca River and its tributaries (Table 7, Figure 2). The opposite was true for aluminum concentrations in water [34]. A similar relationship was found for sediments collected only from the tributaries.

**Figure 3:**
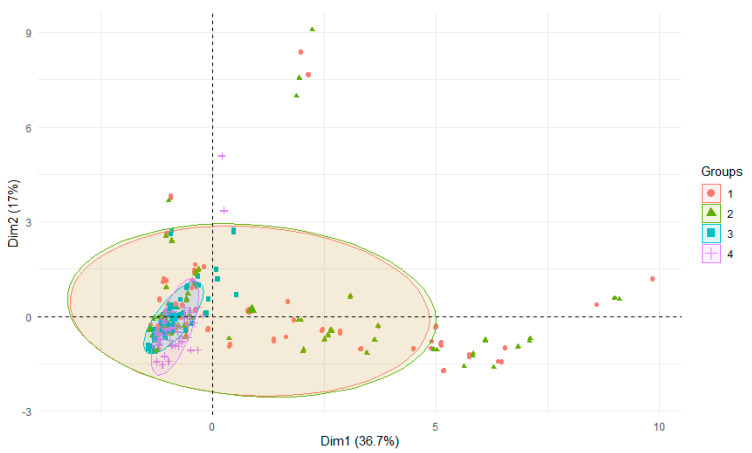
PCA plot 2D showing clustering of years across 29 sites and 3 features (1—2015, 2—2016, 3—2017, 4—2018).

Aluminum content in the sediments of the three mainstem rivers and their tributaries during the four-year study cycle indicates that the levels were similar in the first two years (narrow range of values) and in the last two years (wide range of values) (Figure 3). In the first three study years, Al levels were lower than in the last study year (Table 6). For water, in the last study year (2018), the recorded values were the highest [34]. The reason may be the strong runoff of material from the catchment during intensive rainfall—mainly in spring, which occurred in comparison to the previous study years [41]. In the Nysa Szalona and Strzegomka, the lowest values were found in 2016, and there was no regularity in the case of Bystrzyca.

**Figure 4:**
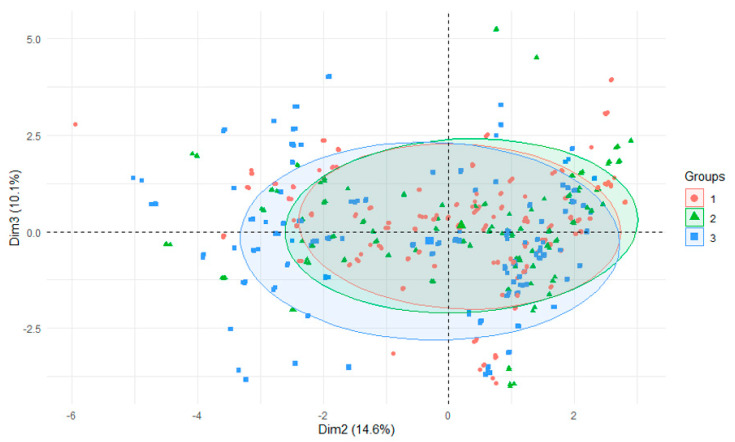
PCA plot 2D showing clustering of rivers across 29 sites and 29 features (1—Bystrzyca, 2—Nysa Szalona, 3—Strzegomka).

**Figure 5:**
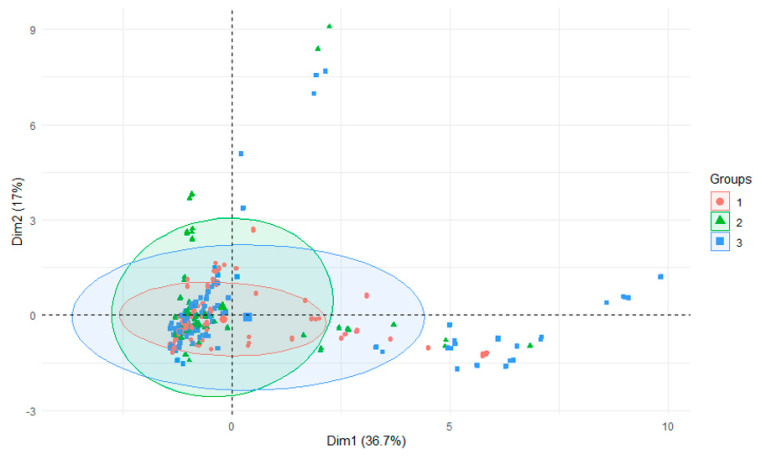
PCA plot 2D showing clustering of section of river across 29 sites and 29 features (1—initial 2—middle, 3—lower section of rivers).

Along the course of the Nysa Szalona and Strzegomka Rivers, among the successive tributaries, initially high aluminum values decreased in subsequent rivers (Table 7). Additionally, within the Strzegomka River, the content of aluminum in sediments decreased with the direction of water flow, with the highest values recorded in the first tributary, Polska Woda, and the lowest in the last one, Czyżynka. On the other hand, in the sediments of the Bystrzyca tributaries it can be seen that in the first four tributaries the level of aluminum in the sediments is lower than in the subsequent ones. All this situation is characteristic for particular rivers and results from the specificity of the catchment. The general picture of the rivers when divided into their initial, middle and lower (estuary to the reservoir) sections, allows to define this level as quite similar, which can also be seen when all parameters are compared (Figure 4). The narrowest range was found for the initial section and the widest for the lower section (Figure 5) [34]. This can also be seen in the overall analysis of all the studied parameters for water and bottom sediments. This allows to conclude that water of varied composition flows into the reservoirs over the entire research cycle, but only a dozen or so years of research could confirm that such a trend is constant [41].

PCA analysis of all parameters in sediments in all rivers depending on initial, middle and lower (estuary to the reservoir) sections of the reservoir.

**Figure 6:**
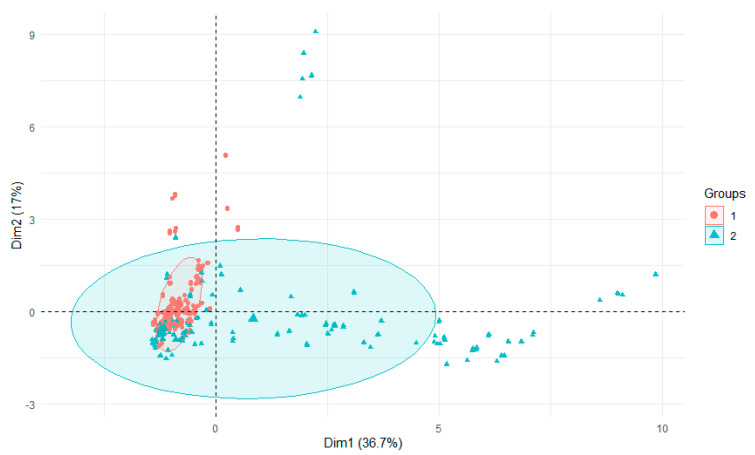
PCA plot 2D showing clustering of season across 29 sites and 3 features (1—spring, 2—autumn).

**Figure 7:**
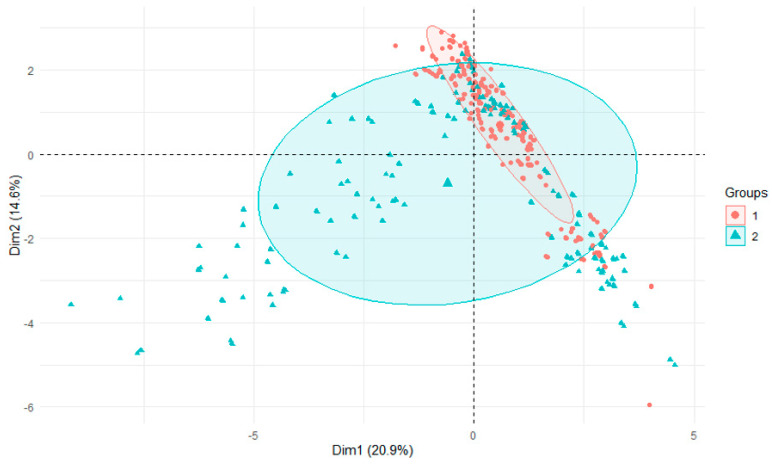
PCA plot 2D showing clustering of season across 29 sites and 29 features (1—spring, 2—autumn).

The variability of the seasons was reflected in the levels of aluminum found in the sediments. In all rivers and their tributaries, autumn values were significantly higher than spring levels, indicating that aluminum was retained in the sediments during the summer (Figure 6). In water, on the other hand, the opposite was true, but here it is probably the result of abundant precipitation and aluminum leaching from the catchment [34]. When all water and sediment parameters were analyzed, autumn values had a wider range of values than spring values (Figure 7) [34]. This may have been influenced by the varying levels of precipitation [41].

A comparison of aluminum contents at two extreme sites, i.e., spring and estuary, on the Nysa Szalona, Bystrzyca and Strzegomka Rivers was made against the background of the seasons of the year (Table 7). It was found that in the Nysa Szalona River in spring and autumn at the sites downstream of the springs the aluminum level was higher than at the estuary to the Słup Reservoir. Such a situation persisted throughout the four-year study period. In the Strzegomka and Bystrzyca Rivers, higher values were recorded in both seasons at the river mouth than below the springs. Against the background of the four-year study period, these two extreme sites showed much higher aluminum contents in the last year of the study, with lower aluminum concentrations in water compared to the remaining years.

Among the tributaries of the Nysa Szalona River, in spring and autumn the highest amounts of aluminum were recorded in the sediments of the first two tributaries (Table 7). No regularity was found for the minimum values. In the Bystrzyca River, the highest amounts of aluminum in spring, in all the years of the study, were found in the sediment taken from Walimianka—the last tributary before the Lubachów Reservoir. In other rivers, lower and similar amounts of aluminum were found. In autumn, the lowest values were recorded in the first four tributaries, while below the level of aluminum was already higher. Within the Strzegomka tributaries, in both seasons of the year, throughout the four-year cycle of the study, in successive tributaries, the content of aluminum decreased, while its concentration in water increased.

**Figure 8:**
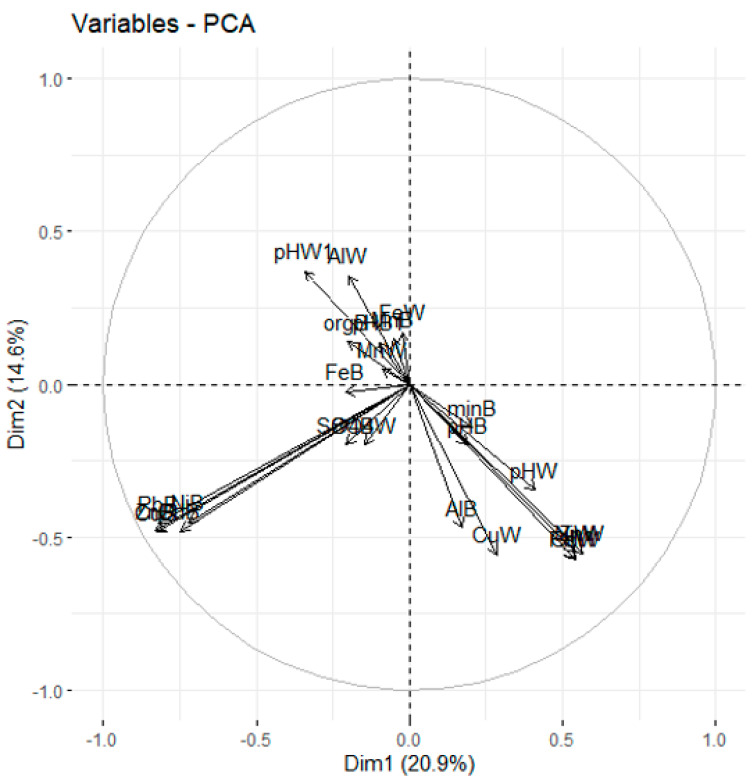
Ordination of the 29 study sites by PCCA based on concentrations of elements in water and sediments in all investigated rivers.

A correlation was found between the level of aluminum in the sediment and the sediment pH, the water pH, the content of mineral compounds and the concentration of metals (Zn, Pb, Cu, Cd) in water, and the concentration of aluminum and sulfates in water (Figure 8) [34]. No correlation was found between aluminum content in water and sediments (Figure 8), which means that the aluminum level in sediments is not dependent on the concentration in water, and therefore it is mainly influenced by the pH and external conditions in the catchment.

The summary of aluminum content on the background of the length of individual rivers does not show any relationship between them. The Strzegomka and Bystrzyca Rivers are of similar length (ca. 20 km) up to their mouths in the reservoirs. The Nysa Szalona, though twice as long, does not contain as much aluminum as the Strzegomka. The size of the catchment area of the river above the reservoir is the largest for the Nysa Szalona, yet it is not reflected in the amount of aluminum carried in the water. In turn, the size of the Strzegomka catchment is the smallest, and the amount of aluminum carried with the waters is the largest. As can be seen, neither the size of the catchment nor the river length for these three rivers have any influence on the level of aluminum in bottom sediments.

In this study, special attention was paid to aluminum compounds deposited in the bottom sediment, classified in the group of substances particularly harmful to the aquatic environment (specific synthetic and non-synthetic pollutants) [42,43]. The accumulation of aluminum and other studied metals in sediments was determined using the concentration factor (CF) [35]. For all metals studied, the lowest values were found in the Strzegomka River, except for Fe and Mn, for which the minimum was in the Bystrzyca River (Table 8). The highest values were found mostly in the Bystrzyca River (Al, Cd, Pb, Zn). In the sediments of the Nysa Szalona River, a higher value was found only for nickel, and cadmium both in Bystrzyca and Nysa was accumulated at a very similar level. In all three main rivers studied, the values recorded in autumn were much higher than in spring (Table 9). In the Strzegomka and Nysa Szalona Rivers, aluminum accumulation at each season was higher at the spring than at the reservoir mouth, and in the Bystrzyca it was the opposite. The same picture was found during the analysis of aluminum content in sediments, which has the main influence on the level of the concentration factor. A comparison of the tributaries against the seasons shows a regularity that is present in all the tributaries—higher values of aluminum concentration factor occurred in autumn.

In order to compare the content of all metals (Al, Cu, Ni, Cd, Pb, Zn, Fe, Mn) in the samples of the three studied rivers and their tributaries, the metal pollution index (MPI) was used [36]. High levels of metal contamination were found for the sediments of the Strzegomka River (Table 10). The highest degree of contamination was observed for the Nysa Szalona and Bystrzyca Rivers. The series of increasing values for sediments within aluminum and heavy metals and metals alone were as follows: Strzegomka < Bystrzyca < Nysa Szalona.

The contamination factor (Cf) was also determined and its lowest values for heavy metals were found in the Strzegomka River (Table 10) [37]. For most metals (Cd, Cu, Pb, Zn) the contamination factor was moderate and for nickel it was significant. For the Nysa Szalona and Bystrzyca, the factor reached a significant level only for cadmium, and for all other metals it was very high. Against the background of heavy metal levels, the level of aluminum was much higher and always described as very high. However, it reached the lowest level in the Strzegomka River, a higher one in the Nysa Szalona River, and the highest one in the Bystrzyca River.

The degree of contamination (DC) was the highest in the Bystrzyca River (26,703.20), lower in the Nysa Szalona River (18,894.73) and lowest in the Strzegomka River (263.60) [37] (Table 10).

The enrichment factor (EF) indicates the influence of anthropogenic pollution on the metal content in the bottom sediments. According to the scale proposed by Sutherland [44], the observed influence of anthropogenic pollution within all the studied sediments was minimal, with a following series of increasing values: Nysa Szalona < Bystrzyca < Strzegomka (Table 10).

The geoaccumulation index (*I_geo_*) was also calculated, which showed that the sediments from the Strzegomka River were the cleanest (class I) [33,38] (Table 11). Against this background, sediments from the Bystrzyca River were more heavily loaded with metals, and those from the Nysa Szalona River were the most polluted.

Taking into account all the indicators of accumulation and contamination of bottom sediments, a clear picture emerges, showing the lowest level for the Strzegomka River (S) and higher levels for the Nysa Szalona River (NS) and the Bystrzyca River (B), respectively (Table 12). On the other hand, the highest level of enrichment factor (EF) indicates soil and catchment effect as the most influential factors on metal levels in the Strzegomka sediment, and the lowest for the Nysa Szalona. The combined picture of all studied parameters in the three main rivers and their tributaries indicates that they are not that far apart, and only their extreme values differentiate them.

Analyses of aluminum in environmental samples against the values quoted for flowing waters in Poland and worldwide show that the type of catchment, the inflow of pollutants and whether the discharged used water undergoes a treatment process are of greatest importance. In the catchments of the studied Lower Silesian rivers, which feed reservoirs serving as drinking water reservoirs, no particularly strong hot spots threatening water quality are recorded. The catchments are typically mountainous, upland and lowland, mainly agricultural but with a prevalence of small farm buildings and small towns. When looking at the variable values in this study, it appears that lower values were found in Poland in samples collected from rivers in north-western Poland, where the range of values was 6.74–47.06 mgAl∙kg^−1^ for the Czerwona River and 3.97–26.15 mgAl∙kg^−1^ for the Grabowa River [45]. The aluminum accumulation coefficients recorded there were also lower in sediments relative to water and amounted for the Czerwona River to 66–23492 and for the Grabowa River to CF = 14–2763. Additionally, in the Insko and Wisola lakes in this region of Poland, the aluminum level in sediments was low [46]. Low values were also recorded in southern Poland in Dziećkowice reservoir and in flowing and standing waters in western Poland [47,48,49].

Samecka-Cymerman et al. [50] also studied aluminum content in bottom sediments from the Nysa Szalona and Strzegomka Rivers and their tributaries. The level found was similar to the present study (2200–6060 mgAl∙kg^−1^). Additionally, in the catchment of the Kamienna River in the Karkonosze region, in an area considered to be unpolluted, the level of aluminum in sediments was similar (3700–5400 mgAl∙kg^−1^) [51]. It is also worth mentioning the rivers flowing in Lower Silesia north of Wroclaw, as well as the lakes of the Legnica and Zielona Góra region, which despite the differentiation of catchment area in terms of pollution load (from low to high) had similar aluminum levels as in this study [52,53,54]. A similar range was also found in Dobromierz Reservoir, into which the Strzegomka River flows, in Piaseczno Reservoir, in the lakes of the Wielkopolski National Park and in Goczałkowicki Lake [55,56,57,58].

Against this background, it is similar in Europe, where the level of aluminum loading of bottom sediments of flowing and standing water bodies varies greatly. A similar range (15,300–54,100 mgAl∙kg^−1^) as in the present study in Poland was found in the Danube River, in highly anthropogenic rivers in Sicily (21,600–26,220 mgAl∙kg^−1^) and in France in the Seine River (1800–5900 mgAl∙kg^−1^) [59,60,61]. Outside the European continent, values similar to those recorded in the present study were also found (India, Cameroon, Nigeria, United States) [62,63,64,65]. Additionally, in industrially treated areas in China, Iran, Egypt, Chile and Mexico, aluminum levels were similar to the present study [66,67,68,69,70,71].

High values for European reservoirs with municipal and commercial catchments that are heavily burdened by heavy industry and mining were recorded in Hungary, Turkey, Russia, Japan, China and Brazil [72,73,74,75,76,77,78].

## 4. Conclusions

The general picture of bottom sediments of the three Lower Silesian rivers shows that they were typically mineral, with neutral pH and moderate sulfate content. The level of aluminum and heavy metals was the highest in the Nysa Szalona and the lowest in the Strzegomka, which was also visible in the concentration factor (CF).

In terms of seasons, higher aluminum contents were recorded in autumn than in spring, which is also reflected in the concentration factor (CF).

Along the course of the river, in the Nysa Szalona and Strzegomka, a gradual decrease in the level of aluminum was observed in successive tributaries, while for the Bystrzyca there was no apparent regularity. Against this background, a comparison of extreme sites below the springs and at the reservoir outlet shows that values were higher in the Nysa Szalona below the springs, and lower in the Strzegomka and Bystrzyca against the background of the reservoir outlet.

The general picture of aluminum and heavy metal loading of the studied sediments shows the lowest loading for the Strzegomka River, only the enrichment factor was the lowest for the Nysa Szalona River: MPI—S < B < NS, C_f_—S < B < NS, DC—S < NS < B, EF—NS < B < S, I_geo_—S < B < NS, CF—S < NS < B.

No influence of catchment size and river length on sediment aluminum levels was found. Attention needs to be focused rather on the sources of pollution present in the catchment, the presence and quality of precipitation, the geological structure, and the modernization works carried out in the riverbed. During them the bank structure is disturbed, new material is introduced and old material is removed. All these works are carried out over quite a long period of time and often cover only fragments of the riverbed but have an impact on water quality and bottom sediments composition.

## Figures and Tables

**Table 1:** MPI value with respect to contamination degree [36].

MPI Value	Contamination Degree
MPI > 2	No influence
2 < MPI < 5	Very low
5 < MPI <10	Low
10 < MPI < 20	Medium
20 < MPI < 50	High
50 < MPI <100	Very high
MPI > 100	The highest

**Table 2:** Contamination factor (C_f_) and degree of contamination (DF) with respect to contamination level [37,38].

Contamination Factor/Degree C_f_ and DC	Contamination Level
C_f_ or DC < 1	Low
1 ≤ C_f_ or DC < 3	Medium
3 ≤ C_f_ or DC < 6	Significant
C_f_ or DC ≥ 6	Very high

**Table 3:** Enrichment factor (EF) [38].

Enrichment Factor (EF)	
EF < 2	No or minimum
2 ≤ EF < 5	Moderate
5 ≤ EF < 20	Significant
20 ≤ EF < 40	Very high
EF > 40	Extremely high

**Table 4:** Geoaccumulation index (*I_geo_*) in relation to the level of bottom sediments contamination [33,38].

Range of *I_geo_* Values	Degree	Level of Bottom Sediments Contamination
<0	0	Uncontaminated
0–1	1	Uncontaminated to moderately contaminated
1–2	2	Moderately contaminated
2–3	3	Moderately to heavily contaminated
3–4	4	Heavily contaminated
4–5	5	Heavily to very heavily contaminated
<5	6	Very heavily contaminated

**Table 5:** Chemical properties of bottom sediments and water of studied rivers [34].

Parameter	Unit	Material	Nysa Szalona	Bystrzyca	Strzegomka
			Min–Max $\bar{x}$ ± SD		
Reaction	pH	B	6.20–8.52 7.18 ± 0.29	6.07–8.54 7.07 ± 0.41	7.01–8.66 7.49 ± 0.41
		W	7.14–8.89 8.12 ± 0.34	7.12–8.68 7.90 ± 0.41	8.01–8.85 8.37 ± 0.22
Mineral compounds	%	B	13.50–99.98 93.64 ± 12.03	24.60–99.90 91.94 ± 13.27	81.00–98.80 95.63 ± 2.92
Organic compounds	%	B	0.02–86.50 6.35 ± 12.03	0.20–75.40 8.05 ± 13.40	1.20–12.30 4.28 ± 2.64
Sulfates	mgSO_4_·kg^−1^	B	3.76–73.16 20.15 ± 13.38	1.42–50.36 11.61 ± 10.60	2.40–62.42 12.17 ± 15.70
	mgSO_4_·dm^−3^	W	7.52–55.68 35.14 ± 11.83	6.28–51.74 28.67 ± 12.35	10.24–50.13 31.49 ± 11.31
Cu	mgCu·kg^−1^	B	6.32–470.96 88.94 ± 123.62	3.28–544.85 62.77 ± 124.23	6.95–17.42 10.98 ± 2.86
	mgCu·dm^−3^	W	0.0024–0.6310 0.0348 ± 0.0454	0.0011–0.2792 0.0246 ± 0.04	0.0020–0.2500 0.0255 ± 0.0383
Ni	mgNi·kg^−1^	B	12.31–1061.55 140.65 ± 212.06	6.37–392.57 54.94 ± 94.11	18.25–26.25 21.20 ± 1.85
	mgNi·dm^−3^	W	0.0001–0.1590 0.0556 ± 0.0584	0.0004–0.3244 0.0329 ± 0.06	0.0024–0.2397 0.0704 ± 0.0691
Cd	mgCd·kg^−1^	B	0.03–10.55 1.79 ± 1.78	0.19–4.98 1.17 ± 0.98	0.45–1.06 0.76 ± 0.16
	mgCd·dm^−3^	W	0.0001–0.0461 0.0102 ± 0.0160	0.0001–0.0014 0.0004 ± 0.0003	0.0001–0.0475 0.0100 ± 0.0167
Pb	mgPb·kg^−1^	B	6.01–569.74 90.72 ± 128.68	8.01–560.36 69.37 ± 112.79	11.02–26.52 17.92 ± 3.67
	mgPb·dm^−3^	W	0.0003–0.3760 0.0779 ± 0.1236	0.0001–0.0098 0.0021 ± 0.0017	0.0005–0.3499 0.0656 ± 0.1153
Zn	mgZn·kg^−1^	B	28.10–2896.41 412.78 ± 646.51	27.22–2782.30 347.75 ± 690.17	31.00–100.21 67.12 ± 18.55
	mgZn·dm^−3^	W	0.0060–1.1657 0.1078 ± 0.1603	0.0015–0.6854 0.0731 ± 0.1240	0.0010–0.6944 0.0927 ± 0.1469
Fe	mgFe·kg^−1^	B	20.41–215,644.90 19,458.21 ± 21,538.07	88.55–18,244.70 9299.19 ± 4400.21	230.14–19,632.41 10,289.74 ± 5994.02
	mgFe·dm^−3^	W	0.0862–4.7623 0.8505 ± 0.6901	0.0765–2.6359 1.0391 ± 0.5000	0.2312–1.7388 0.6195 ± 0.3401
Mn	mgMn·kg^−1^	B	96.23–876.91 174.85 ± 86.73	4.34–597.43 105.85 ± 92.93	7.74–212.63 116.41 ± 62.85
	mgMn·dm^−3^	W	0.0112–0.9786 0.2081 ± 0.2013	0.0536–0.9675 0.2893 ± 0.2008	0.0123–0.9652 0.2617 ± 0.2002

Sediments—B, Water—W; Min—minimum value, max—maximum value, $\bar{x}$—average value, SD—standard deviation.

**Table 6:** Aluminum content in bottom sediments (mgAl·kg^−1^) and water (mgAl·dm^−3^) depending on the year [34].

Site/Material			2015	2016	2017	2018
			Min–Max $\bar{x}$ ± SD	Min–Max $\bar{x}$ ± SD	Min–Max $\bar{x}$ ± SD	Min–Max $\bar{x}$ ± SD
Nysa Szalona	below springs	B	3841.20–5623.11 4732.86 ± 889.62	1562.36–4290.03 2926.24 ± 1363.52	3970.90–4453.22 4211.88 ± 240.68	4018.47–76,287.69 40152.61 ± 36,133.81
		W	0.1382 ± 0.03	0.1311 ± 0.03	0.1940 ± 0.02	0.0767 ± 0.02
	tributaries	B	1024.95–32,345.21 7121.49 ± 7757.60	1425.04–34,465.11 7422.01 ± 8206.89	1412.78–5743.78 2994.52 ± 1042.82	1264.96–96,260.32 31,664.04 ± 30,309.50
		W	0.1717 ± 0.06	0.1388 ± 0.04	0.2508 ± 0.07	0.0871 ± 0.01
	reservoir outlet	B	1896.05–2696.30 2290.47 ± 394.22	1345.33–2638.50 1991.57 ± 646.07	2832.10–2900.77 2868.26 ± 32.32	2563.09–69,102.33 35,832.50 ± 33,269.07
		W	0.2004 ± 0.01	0.1565 ± 0.04	0.3093 ± 0.01	0.0906 ± 0.01
Strzegomka	below springs	B	14.24–147.52 80.79 ± 66.49	13.46–125.48 69.50 ± 55.97	15.38–256.32 135.93 ± 120.31	15.03–468.98 241.96 ± 226.62
		W	0.1544 ± 0.02	0.1106 ± 0.01	0.1643 ± 0.02	0.1067 ± 0.01
	tributaries	B	25.52–256.36 132.10 ± 105.53	18.18–196.28 89.47 ± 70.81	18.33–369.54 159.67 ± 145.44	28.05–621.47 307.89 ± 277.05
		W	0.1711 ± 0.02	0.1416 ± 0.02	0.1814 ± 0.02	0.1963 ± 0.06
	reservoir outlet	B	15.32–159.74 87.47 ± 72.08	16.85–123.45 70.14 ± 53.25	15.43–456.33 235.86 ± 220.36	25.34–569.88 297.51 ± 272.04
		W	0.1651 ± 0.01	0.1391 ± 0.02	0.1700 ± 0.01	0.1891 ± 0.01
Bystrzyca	below springs	B	2861.41–26,479.32 14,670.23 ± 11,808.46	2636.43–25,064.11 13,850.35 ± 11,213.26	2103.52–24,863.01 13,483.12 ± 11,379.58	2706.07–63,958.45 33,332.34 ± 30,625.58
		W	0.1744 ± 0.01	0.1724 ± 0.01	0.1951 ± 0.01	0.1166 ± 0.02
	tributaries	B	1701.22–21,202.67 9070.44 ± 7653.74	1632.65–85,370.32 14,139.50 ± 20,118.86	1503.32–21,631.44 10,759.78 ± 8501.23	2445.3–90,318.42 33,483.67 ± 32,232.75
		W	0.1315 ± 0.03	0.1429 ± 0.02	0.1489 ± 0.02	0.1185 ± 0.01
	reservoir outlet	B	2706.07–16,292.22 9499.29 ± 6791.95	2610.5–16,925.32 9768.32 ± 7156.79	5004.64–49,210.13 27,107.64 ± 22,102.44	2354.78–61,563.22 31,959.04 ± 29,603.92
		W	0.1038 ± 0.02	0.1296 ± 0.01	0.1647 ± 0.02	0.1087 ± 0.01

Sediments—B, water—W; Min—minimum value, max—maximum value, $\bar{x}$—average value, SD—standard deviation.

**Table 7:** Aluminum content in bottom sediments (mgAl·kg^−1^) and water (mgAl·dm^−3^) of rivers in spring and autumn [34].

Site (No.)	Material	Nysa Szalona		Strzegomka		Bystrzyca	
		Spring	Autumn	Spring	Autumn	Spring	Autumn
		Min–Max $\bar{x}$ ± SD					
1	B	3841.20–4453.22 4151.09 ± 235.79	1562.36–76,287.69 21,860.71 ± 31,455.85	13.46–15.78 14.70 ± 0.85	125.47–468.98 249.39 ± 135.90	2103.52–2862.11 2577.29 ± 285.35	24,862.40–63,958.45 35,090.73 ± 16,678.11
	W	0.0601–0.6020 0.1789 ± 0.1391	0.0930–0.1738 0.1351 ± 0.03	0.1122–0.1863 0.1468 ± 0.03	0.1004–0.1433 0.1200 ± 0.02	0.1365–0.1966 0.1736 ± 0.02	0.0978–0.1945 0.1564 ± 0.04
2	B	2501.71–2698.12 2637.29 ± 79.75	2596.44–65,325.00 33,705.72 ± 22,192.65	25.36–32.33 29.61 ± 2.47	112.01–621.47 339.34 ± 186.60	2046.30–3025.44 2339.43 ± 399.03	20,144.56–45,628.12 26,780.34 ± 10,889.51
	W	0.0801–0.2367 0.1570 ± 0.0735	0.0850–0.1538 0.1294 ± 0.0266	0.1233–0.1987 0.1538 ± 0.0302	0.1123–0.1555 0.1326 ± 0.0177	0.1244–0.1856 0.1594 ± 0.03	0.0990–0.1897 0.1499 ± 0.03
3	B	5501.25–6015.52 5684.26 ± 206.86	2325.41–96,260.32 26,022.62 ± 40,551.81	25.09–35.98 28.03 ± 4.48	196.25–533.74 327.80 ± 127.20	2054.11–3055.18 2441.35 ± 372.00	10,151.40–73,024.10 27,969.10 ± 26,174.40
	W	0.0902–0.2137 0.1483 ± 0.0555	0.0920–0.1451 0.1246 ± 0.0199	0.1625–0.2247 0.1966 ± 0.0240	0.1585–0.2154 0.1890 ± 0.0202	0.1152–0.1634 0.1443 ± 0.02	0.1201–0.1746 0.1382 ± 0.02
4	B	2036.55–2411.26 2244.95 ± 138.14	2481.72–44,026.21 19,070.39 ± 15271.86	18.18–29.36 23.61 ± 5.23	159.23–596.54 285.31 ± 180.07	1750.16–2641.96 2041.01 ± 351.11	18,551.34–50,457.40 27,511.12 ± 13,290.93
	W	0.0874–0.2243 0.1532 ± 0.0623	0.0908–0.1745 0.1310 ± 0.0316	0.1452–0.2589 0.1951 ± 0.04	0.1422–0.2489 0.1702 ± 0.04	0.1235–0.1591 0.1429 ± 0.01	0.1100–0.1607 0.1279 ± 0.02
5	B	3574.10–4013.52 3738.31 ± 176.29	1324.12–54,226.12 14,935.27 ± 22,688.50	15.32–25.63 18.31 ± 4.18	123.32–569.88 327.18 ± 190.41	1845.20–2853.90 2176.09 ± 407.22	12,569.50–53,622.00 26,604.49 ± 15,886.33
	W	0.0813–0.2902 0.1659 ± 0.0875	0.0820–0.1797 0.1365 ± 0.0371	0.1527–0.1954 0.1751 ± 0.01	0.1232–0.1863 0.1575 ± 0.02	0.1236–0.1598 0.1362 ± 0.01	0.0933–0.1525 0.1227 ± 0.03
6	B	1815.53–2212.73 1992.29 ± 145.44	1412.78–53,935.90 18,623.67 ± 20,660.00			1780.49–3288.34 2270.24 ± 594.31	18,225.21–85,370.32 47,384.77 ± 29,693.91
	W	0.0130–0.3055 0.1745 ± 0.0963	0.0861–0.1687 0.1405 ± 0.0336			0.1342–0.1647 0.1447 ± 0.01	0.0820–0.1323 0.1088 ± 0.02
7	B	2872.21–3200.36 3010.70 ± 130.07	1526.33–69,514.68 187,17.97 ± 29,328.48			1875.67–2910.45 2402.17 ± 501.63	10,634.50–90,318.42 32,853.50 ± 33,361.22
	W	0.0824–0.2641 0.1774 ± 0.0752	0.0933–0.2544 0.1501 ± 0.0635			0.1253–0.1936 0.1512 ± 0.03	0.1159–0.1335 0.1242 ± 0.01
8	B	2455.01–2636.90 2543.19 ± 75.77	2623.67–53,346.65 22,878.27 ± 18,648.05			1503.32–2534.66 1843.50 ± 404.87	16,330.00–75,632.88 32,717.70 ± 24,810.44
	W	0.0917–0.2451 0.1389 ± 0.0621	0.0914–0.3691 0.1846 ± 0.1087			0.1026–0.1456 0.1270 ± 0.02	0.1125–0.1378 0.1263 ± 0.10
9	B	2285.20–2897.67 2595.09 ± 222.80	2446.54–70,098.33 23,874.99 ± 26,940.52			2445.30–4087.66 2890.99 ± 690.81	18,941.20–56,945.24 29,587.68 ± 15,884.29
	W	0.0915–0.2197 0.1557 ± 0.0457	0.0810–0.3782 0.1900 ± 0.1134			0.1054–0.1464 0.1331 ± 0.02	0.1258–0.1432 0.1324 ± 0.01
10	B	1024.95–4565.93 2070.21 ± 1447.09	2010.45–63,285.00 23,108.42 ± 23,669.93			2354.78–5005.84 3169.79 ± 1067.47	16,290.30–61,563.22 35,997.35 ± 19,876.23
	W	0.0034–0.2121 0.1402 ± 0.0811	0.0950–0.3460 0.1877 ± 0.0945			0.1053–0.1865 0.1371 ± 0.03	0.0860–0.1407 0.1152 ± 0.02
11	B	2088.22–2596.30 2283.90 ± 188.50	2349.01–55,204.89 19,707.84 ± 20,775.31				
	W	0.0915–0.3653 0.1659 ± 0.1153	0.0911–0.2894 0.1724 ± 0.0727				
12	B	3014.59–3504.07 3195.35 ± 198.70	1525.33–47,998.32 13,670.63 ± 19,835.86				
	W	0.0946–0.3538 0.1779 ± 0.1027	0.0986–0.3356 0.2086 ± 0.0955				
13	B	2024.78–3915.34 3243.39 ± 510.43	5733.20–52,250.34 25,657.67 ± 16,779.95				
	W	0.0917–0.1951 0.1349 ± 0.0379	0.0825–0.3446 0.2289 ± 0.0940				
14	B	2563.09–2843.68 2680.47 ± 99.86	1345.33–69,102.33 18,810.93 ± 29,040.66				
	W	0.1770 ± 0.08	0.2007 ± 0.08				
Tributaries together sediments		1024.95–6015.52 2936.58 ± 1075.84	1324.12–96,260.32 21,664.46 ± 24,560.21	18.18–35.98 27.08 ± 4.93	112.01–621.47 317.48 ± 168.37	1503.32–4087.66 2300.60 ± 559.64	10,151.40–90,318,42 31,426.09 ± 23,529.62
		1024.95–96,260.32 12,300.52 ± 19744		18.18–621.47 172.28 ± 187.80		1503.32–90,318.42 16,863.34 ± 22,114.51	
Tributaries together water		0.0034–0.3653 0.1588 ± 0.08	0.0810–0.3782 0.1654 ± 0.08	0.1233–0.2589 0.1818 ± 0.04	0.1123–0.2489 0.1634 ± 0.04	0.0990–0.1936 0.1420 ± 0.02	0.0820–0.1897 0.1289 ± 0.02
		0.0034–0.3782 0.1614 ± 0.08		0.1123–0.2589 0.1729 ± 0.04		0.0820–1936 0.1356 ± 0.02	
Water in total		0.0034–0.6020 0.1621 ± 0.08		0.1004–0.2589 0.1637 ± 0.04		0.0820–0.1966 0.1355 ± 0.02	
Sediments in total		1024.95–96,260.32 12,239.84 ± 20,250.46		13.4587–621.47 164.33 ± 185.53		1503.32–90,318.42 17,332.43 ± 21,891.58	

Sediments—B, water—W; min—minimum value, max—maximum value, $\bar{x}$—average value, SD—standard deviation.

**Table 8:** Concentration factor (CF) of aluminum and heavy metals in bottom sediments.

	Nysa Szalona	Bystrzyca	Strzegomka
Al.	75,045.00	125,962.40	1003.83
Cu	2555.72	2551.48	430.47
Ni	2529.75	1669.80	301.12
Cd	175.50	2930.00	76.33
Pb	1164.56	32,898.90	273.19
Zn	3829.10	4757.10	724.08
Mn	840.21	365.88	444.82
Fe	22,878.55	8949.27	16,610.75

**Table 9:** Concentration factor (CF) of aluminum in bottom sediments depending on the season.

	Season	Nysa Szalona	Bystrzyca	Strzegomka
In total	spring	29,761.74	134.59	17,091.9
	autumn	210,152.61	2002.42	266,764.5
Tributaries	spring	30,334.33	154.57	16,622.68
	autumn	211,273.76	1952.51	260,853
Below springs	spring	33,861.22	102.12	15,235.28
	autumn	225,544.67	2193.72	272,468.6
Reservoir outlet	spring	18,791.21	104.14	22,702.22
	autumn	308,809.04	1960.86	308,352.3

**Table 10:** Metal pollution index (MPI), contamination factor (C_f_), degree of contamination (DC) and enrichment factor (EF) of bottom sediments with metals.

	Nysa Szalona				Bystrzyca				Strzegomka			
	MPI	C_f_	DC	EF	MPI	C_f_	DC	EF	MPI	C_f_	DC	EF
Al	277.26	18,830.53	18,896.06	-	189.41	26,665.28	26,703.91	-	50.46	252.81	264.35	-
Cd		3.58		0.0002		2.34		0.0001		1.53		0.0060
Cu		14.82		0.0001		10.46		0.0004		1.83		0.0072
Ni		28.14		0.0015		10.99		0.0004		4.24		0.0168
Pb		9.07		0.0005		6.91		0.0003		1.79		0.0071
Zn		8.59		0.0005		7.24		0.0003		1.40		0.0055
Mn		0.35		0.0001		0.46		0.00001		0.23		0.0017
Fe		0.97		0.0217		0.21		0.2812		0.51		0.0040

**Table 11:** Geoaccumulation index (*I_geo_*) and purity class of bottom sediments.

	Nysa Szalona		Bystrzyca		Strzegomka	
	I_geo_	Class	I_geo_	Class	I_geo_	Class
Cu	3	III	2	II	0	I
Ni	4	III	2	II	1	II
Cd	1	II	0	I	0	I
Pb	2	II	2	II	0	I
Zn	2	II	3	III	0	I
Mn	0	I	0	I	0	I
Fe	0	I	0	I	0	I

**Table 12:** Comparison of contamination indices.

MPI	S < B < NS
C_f_	S < B < NS
DC	S < NS < B
EF	NS < B < S
I_geo_	S < B < NS
CF	S < NS < B

## Data Availability

Not applicable.
